# Molecular Characterization and Growth Association of Two Apolipoprotein A-Ib Genes in Common Carp (*Cyprinus carpio*)

**DOI:** 10.3390/ijms17091569

**Published:** 2016-09-16

**Authors:** Xinhua Wang, Xiaomu Yu, Jingou Tong

**Affiliations:** 1 State Key Laboratory of Freshwater Ecology and Biotechnology, Institute of Hydrobiology, the Chinese Academy of Sciences, Wuhan 430072, China; xinhuawang123@163.com (X.W.); xmyu@ihb.ac.cn (X.Y.); 2 Graduate School, University of Chinese Academy of Sciences, Beijing 100049, China

**Keywords:** *Cyprinus carpio*, apolipoprotein A-I, gene expression, compensatory growth, growth association

## Abstract

Apolipoprotein A-I (ApoA-I) is functionally involved in the transportation and metabolism of lipids in vertebrates. In this study, two isoforms of *apoA-Ib* in common carp (*Cyprinus carpio* L.) were characterized. Sequence comparison and phylogenetic analysis showed that *C*. *carpio* ApoA-Ib is relatively conserved within cyprinid fishes. During embryonic development, *C. carpio* *apoA-Ib* was first expressed at the stage of multi-cells, and the highest mRNA level was observed at the stage of optic vesicle. A ubiquitous expression pattern was detected in various tissues with extreme predominance in the liver. Significantly different expression levels were observed between light and heavy body weight groups and also in the compensatory growth test. Seventeen and eight single-nucleotide polymorphisms (SNPs) were identified in matured mRNA of the *C. carpio* *apoA-Ib.1* and *apoA-Ib.2*, respectively. Two of these SNPs (apoA-Ib.2-g.183A>T and apoA-Ib.2-g.1753C>T) were significantly associated with body weight and body length in two populations of common carp. These results indicate that *apoA-Ib* may play an important role in the modulation of growth and development in common carp.

## 1. Introduction

Apolipoproteins A-I (ApoA-I), as one of the nine major apolipoproteins [1], plays an important role in the transportation and metabolism of lipids [2,3,4]. Besides this, ApoA-I also participates in a number of processes beyond lipid transport, such as its ability to inhibit inflammation and as an antioxidant [5,6,7]. Studies on the *apoA-I* gene, including gene structure, expression and function analysis, have been performed in humans and animals during the past few years [3,8,9,10].

Fish are poikilothermal animals and use lipids as their main energy source; thus, lipoproteins may have unique roles in fish compared with other vertebrates [11,12]. Kondo et al. [12] pointed out that a low homology existed between fish apolipoproteins and their counterparts in mammals. The tissue expression analyses of *apoA-I* in some fish species showed that the liver was always observed with the highest expression, but more variable expression levels were detected in the intestine, gills and epidermis [13,14,15,16,17,18]. The antibacterial properties of ApoA-I have also been detected in several fish species [17,18,19]. However, few studies on growth-related functions of ApoA-I had been reported in fish [20].

Common carp (*Cyprinus carpio* L.), as one of the dominant cyprinid species, is widely distributed in Asia and Europe with a domestication history of over two thousand years [21]. Most teleosts had undergone the teleost-specific genome duplication (TSGD), and an additional whole-genome duplication (WGD) event tetraploidized the genome of *Cyprinus carpio* [22,23]. Isoforms of *apoA-I* have been identified in some cyprinid fish, such as *Danio rerio* [24], *Cyprinus carpio* [18], *Anguilla japonica* [25], *Oncorhynchus mykiss* [26] and *Hemibarbus mylodon* [27]. Complete genomic sequences of two *apoA-Ibs* of common carp are available from the GenBank database (*apoA-Ib.1* and *apoA-Ib.2*; Accession Nos. KJ741859 and KJ741860); however, no characterization information is available, and whether *C.* *carpio* *apoA-Ibs* associate with growth traits remains unclear.

The present study aims to characterize the two *apoA-Ib* genes of common carp and to illustrate their spatial and temporal expression patterns. The expression analysis of *apoA-Ib* was performed in different growth tests of common carp. The growth associations were also performed for single nucleotide polymorphisms (SNPs) in *apoA-Ib.1* and *apoA-Ib.2*. This study would contribute to understanding the biological function of the *apoA-Ib* in common carp.

## 2. Results

### 2.1. Characterization of apoA-Ibs

**Figure 1:**
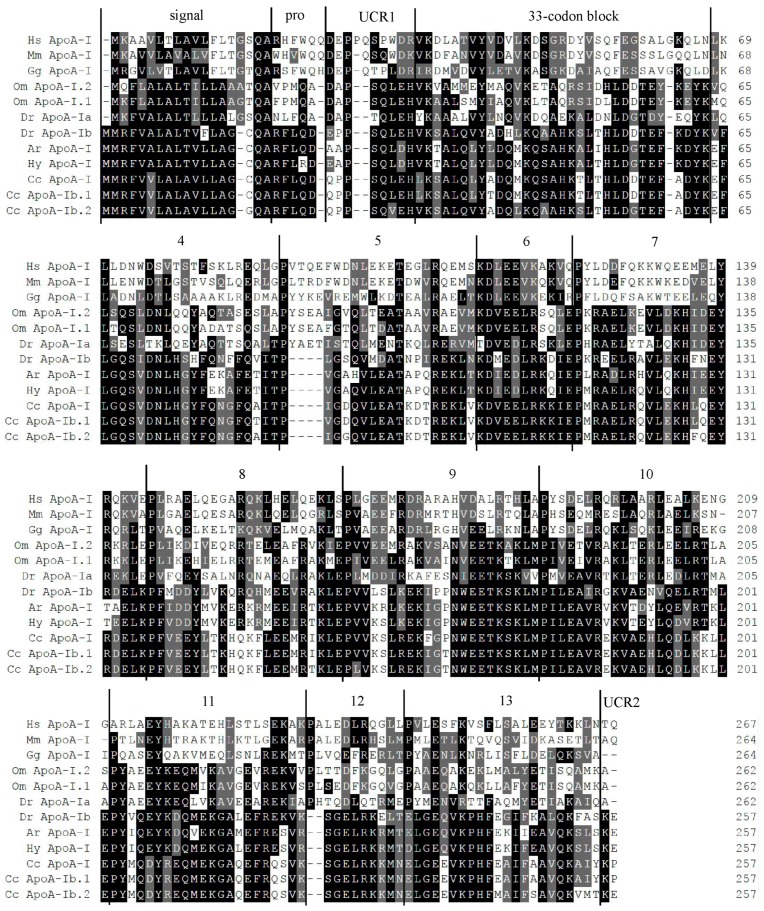
Comparison of deduced amino acid sequences of *C. carpio* ApoA-Ibs with several other species of vertebrates. Their accession numbers are as follows: Cc-ApoA-Ib.1 (*Cyprinus carpio*, KJ741859), Cc ApoA-Ib.2 (*Cyprinus carpio*, KJ741860), Cc ApoA-I (*Cyprinus carpio*, KF268349), Ar ApoA-I (*Hypophthalmichthys* *nobilis*, unpublished result), Hy ApoA-I (*Hypophthalmichthys molitrix*, ADF97611), Dr ApoA-Ib (*Danio rerio*, NP_001093614), Dr ApoA-Ia (*Danio rerio*, NP_571203), Om ApoA-I.1 (*Oncorhynchus mykiss*, NP_001117719), Om ApoA-I.2 (*Oncorhynchus mykiss*, NP_001117720), Gg ApoA-I (*Gallus gallus*, NP_990856), Mm ApoA-I (*Mus Musculus*, NP_033822), Hs ApoA-I (*Homo sapiens*, NP_000030). Boundaries of the signal peptide (signal), the propeptide (pro), the unrelated coding regions 1 and 2 (UCR1 and UCR2, respectively), the 33-codon block and the 11- or 22-residue repeats (4 to 13) are indicated above the sequence of Hs ApoA-I. Identity is covered with black and grey color.

Two *C.* *carpio* *apoA-Ibs* both contain three exons, two introns and an extra intron in 5’-untranslated regions (5’-UTR). Comparison of nucleotide sequences between two subtypes of *C.* *carpio apoA-Ib* showed a higher variation in introns (75.3% identity) and a lower difference in exons (96.5% identity; Figure S1) with twenty-one amino acid changes (Figure 1). The sequences of mature *C.* *carpio* ApoA-Ibs both satisfy typical common structural features of ApoA-I, including an 18-amino acid-long signal peptide, a 5-amino acid-long prosegment, two unrelated coding regions, a 33-codon block and ten 11- or 22-residue repeats (Figure 1). The comparison of amino acid sequences revealed that ApoA-I was less conserved throughout the vertebrates. Specifically, *C.* *carpio* ApoA-Ibs shared a higher homology with *Cyprinus carpio* ApoA-I (97%), *Hypophthalmichthys molitrix* ApoA-I (74%), *Danio rerio* ApoA-Ib (71%), *Hypophthalmichthys* *nobilis* ApoA-I (70%) and a lower homology with *Oncorhynchus mykiss* ApoA-I.1 and ApoA-I.2 (42%), *Danio rerio* ApoA-Ia (41%), *Gallus gallus* ApoA-I (28%), *Mus musculus* ApoA-I (25%) and *Homo sapiens* ApoA-I (26%).

**Figure 2:**
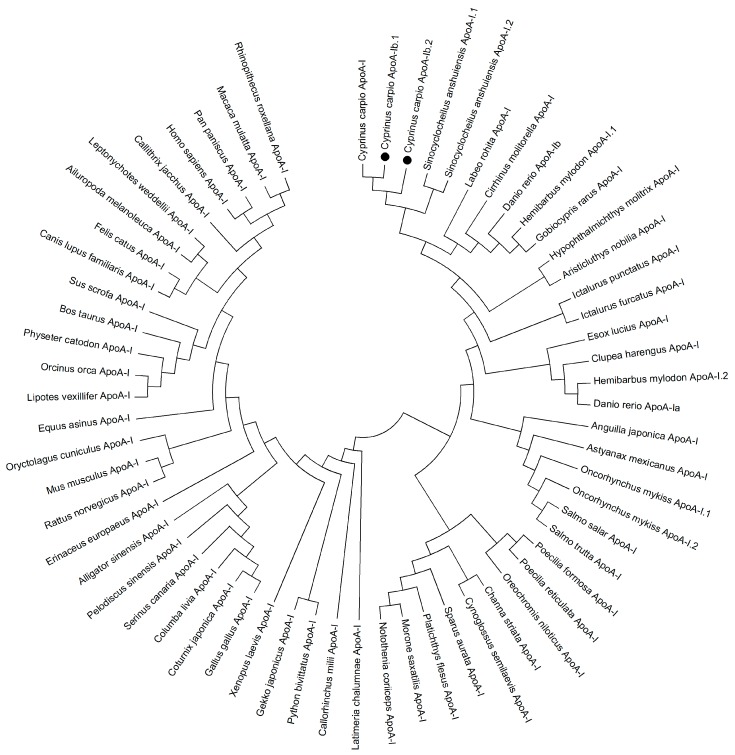
Neighbor-joining phylogenetic tree based on 63 ApoA-I protein sequences of vertebrates. *Cyprinus* *carpio* ApoA-Ib.1 and ApoA-Ib.2 were marked by bold dot.

A phylogenetic tree based on 63 ApoA-I protein sequences (Table S1) demonstrated a pattern of two subgroups in vertebrates (Figure 2), with the one including teleosts and the other including mammalian, avian, reptilia, amphibians and chondrichthyes. The ApoA-I of most species in the Cypriniformes, including *C.* *carpio* ApoA-Ibs, were clustered together with ApoA-Ib of zebrafish; however, ApoA-Ia of zebrafish was clustered to another group of fish in the Siluriformes.

### 2.2. Expression of C. carpio apoA-Ib

**Figure 3:**
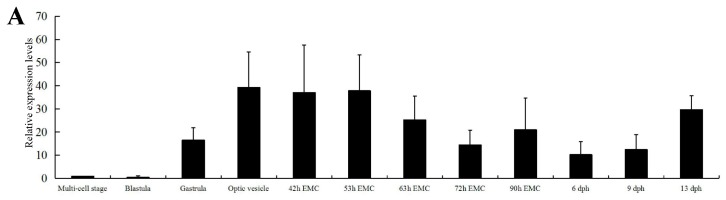

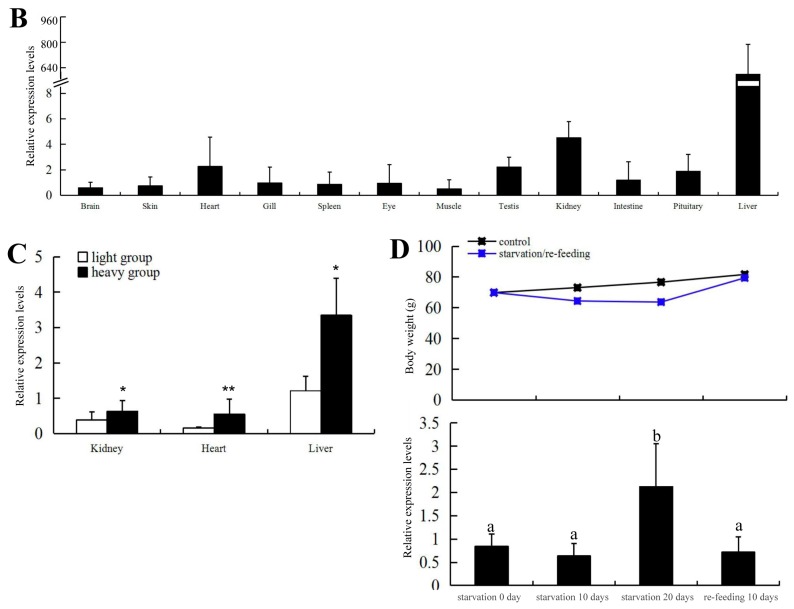
The relative expression levels of *apoA-Ib* in *Cyprinus carpio*. (**A**) The relative expression during embryonic development; (**B**) The relative expression in different tissues; (**C**) Comparative expression analysis of kidney, heart and liver between light and heavy body weight groups. Significant differences at *p* < 0.05 and *p* < 0.01 are labeled with * and **, respectively; (**D**) Body weight and expression analysis of the individuals participated in the compensatory growth test. Significant differences are labeled with different lowercase letters. Data are shown as the mean ± SEM (*n* = 5).

During embryonic development, an expression pattern of rapid increase then gradual decrease was observed for *C. carpio* a*poA-Ib*, and the highest expression level was detected at the stage of optic vesicle (Figure 3A). *C. carpio* a*poA-Ib* showed a ubiquitous expression pattern in all tissues analyzed, and the highest level was observed in the liver, followed by the kidney, heart and testis (Figure 3B). Three tissues (the liver, heart and kidney) with higher expression levels were selected for comparative analysis between light and heavy body weight groups. Significantly different expression levels between two weight groups were detected in the heart and liver (*p* < 0.01 and *p* < 0.05, respectively), but not in the kidney (Figure 3C). In the compensatory growth test, the liver was selected for the expression analysis of *C. carpio apoA-Ib*, in which the expression level was much higher than other tissues analyzed. The body weights of the fish were decreased during starvation and approached the control level after 10 days of re-feeding (Figure 3D). Compared with starvation 0 day, the expression level was significantly increased at starvation 20 days, but not at starvation 10 days. After re-feeding 10 days, the expression level decreased to the control level (Figure 3D).

### 2.3. Polymorphisms of the C. carpio apoA-Ibs

Large numbers of putative SNPs were identified in *C. carpio apoA-Ibs* based on multiple sequence alignments. The SNPs were widely distributed in the genomic sequences of *C. carpio* a*poA-Ib.1* and *apoA-Ib.2*, but only those SNPs in the UTRs or exons were used for SNP genotyping in this study. Finally, a total of 25 SNPs (seventeen for *apoA-Ib.1* and eight for *apoA-Ib.2*, respectively) were genotyped using 100 individuals of Yellow River carp. Detailed information for the parameters of genetic diversity are listed in Table 1. The mean values of the observed and expected heterozygosities (*H_O_* and *H_E_*) were 0.324 and 0.320, respectively. After Bonferroni corrections, two polymorphic loci g.1746C>T and g.2009C>G in *C. carpio* a*poA-Ib.1* were significantly deviated from the Hardy-Weinberg equilibrium (HWE). Loci g.1687T>A, g.1689T>C and g.1693A>C in *C. carpio* a*poA-Ib.1* were completely linked, and loci g.1961G>C and g.1966G>C in *apoA-Ib.1* were linked, as well.

### 2.4. Genetic Associations between SNPs and Growth Traits

Three polymorphic loci with growth associations in the first one hundred individuals of Yellow River carp (apoA-Ib.1-g.1693A>C, apoA-Ib.2-g.183A>T and apoA-Ib.2-g.1753C>T) were additionally genotyped by PCR-restriction fragment length polymorphism (PCR-RFLP) in the remaining 100 individuals. After association analysis for all 200 individuals, no significant association was detected between growth traits and the locus apoA-Ib.1-g.1693A>C, while the loci g.183A>T and g.1753C>T in *apoA-Ib.2* were still significantly associated with growth traits (Table 2). In detail, apoA-Ib.2-g.183A>T showed highly significant association with body length (BL) and body weight (BW) (*p* < 0.01), and the individuals with genotype AT had a higher BW (6.5%) than the individuals with genotype AA. Significant associations were also observed between apoA-Ib.2-g.1753C>T and BL (*p* < 0.05) and BW (*p* < 0.01), and individuals of the genotype CC had a higher value of BW compared with those with genotype CT.

Another population that contains 200 individuals of Yangtze River carp was used for further verification of growth associations at loci g.183A>T and g.1753C>T in *C. carpio* *apoA-Ib.2*. Although only two genotypes were detected for the two loci in this population, significant associations were also observed (Table 2). For the locus apoA-Ib.2-g.183A>T, significant association with BW (*p* < 0.05) was detected, and the individuals with genotype AT had a higher BW (16.4%) than individuals with genotype AA. The locus apoA-Ib.2-g.1753C>T was extremely significantly associated with BL and BW (*p* < 0.01), and the average BW of those individuals with genotype CC was 6.5% higher than that with genotype CT.

## 3. Discussion

As one of the major HDL proteins, ApoA-I has been extensively studied for lipid transport and anti-inflammation properties in mammals [5,7,28,29]. A number of studies on *apoA-I* have also been reported for aquatic animals, and the functional studies were mainly focused on lipid metabolism and diverse protective pathways [17,18,30,31,32]. Two isoforms of *apoA-I* (*apoA-Ia* and *apoA-Ib*) were detected for zebrafish in recent years [24], coupled with the latest round of the whole-genome duplication (WGD) event, which was estimated to occur 8.2 million years ago and resulted in tetraploidization of the *Cyprinus carpio* genome [23]; therefore, the existence of two isoforms of *apoA-Ib* in common carp is likely reasonable. Interestingly, the genomic sequences of *C. carpio* *apoA-Ib.1* and *apoA-Ib.2* were positioned onto two non-homologous linkage groups (LG36 and LG13, respectively) of common carp genome [23]. These results may suggest that the *C. carpio* *apoA-Ib.1* and *apoA-Ib.2* evolved from a common gene and experienced chromosome rearrangements after gene duplication.

In our study, the amino acid sequences of *C. carpio* ApoA-Ibs showed low homology with their mammalian counterparts (26% identity; Figure 1). A similar phenomenon was also detected for *Takifugu rubripes* ApoA-I (21% identity) [12], *Danio rerio* ApoA-I (25.6% identity) [33], *Oncorhynchus mykiss* ApoA-I.1 and ApoA-I.2 (28% identity) [28] and *Cyprinus carpio* ApoA-I (27% identity) [18]. Expression patterns of *apoA-I* have been reported in several fish species [13,14,15,16,17,25,33], but studies on temporal and spatial expression patterns of *apoA-I* were not performed in common carp. The expression pattern of *apoA-Ib* in embryonic development of common carp (Figure 3A) was similar to that of zebrafish [33] and turbot [34]. The high expression level before hatching may indicate that *C. carpio apoA-Ib* is more likely to be related to the transference of structural lipids than to lipids used for energy purposes at the early development stages [33,34]. The tissue expression patterns of *apoA-I* in fishes analyzed, including common carp in our study, were not completely consistent with each other (Figure 3B) [13,14,15,16,24,25,27], which confirmed the conclusion that the tissue expression patterns of the apolipoprotein were species-specific [12,26]. Concha et al. [16] claimed that carp may have an unusual mechanism of dietary lipid because of the undetectable expression of *apoA-I* in the intestine. However, the intestine is generally proven to be an organ that expressed *apoA-I* in fish [12,13,14,17,27] and is no exception in our study. The undetectable expression of *apoA-I* in intestine published by Concha et al. [16] may be due to insensitive detection methods, such as Northern blot and RT-PCR.

Significantly different expression levels of *apoA-Ib* before and after re-feeding and between light and heavy groups of common carp in this study (Figure 3C,D) may indicate that *apoA-Ib* plays an important role in the regulation of growth. The increased expression of *C. carpio* *apoA-Ib* at starvation 20 days may suggest that fish need to consume their own lipids to maintain normal metabolism during fasting. Therefore, the fish synthesize much more ApoA-Ib proteins to transport the lipids during fasting and convert it to absorb energy from external food after re-feeding. A similar phenomenon for the increased expression level of *apoA-I* during fasting had been observed in different human populations [35,36,37]. Additionally, available growth association of *apoA-I* in grass carp [20] further indicated that *apoA-I* may be involved in the regulation of growth.

The candidate gene approach provides a powerful tool to study the genetic architecture of complex quantitative traits in aquatic animals [38,39]. The association study between specific alleles of single-nucleotide polymorphisms (SNPs) detected within a candidate gene and the trait of interest is a common strategy to elucidate quantitative trait nucleotides (QTN) and major genes that may affect quantitative polygenic traits, and the method has been successfully applied to several growth candidate genes in common carp [40,41,42]. Compared with grass carp [20], large numbers of SNPs (Table 1) in the present study may indicate that the polymorphic levels of *C. carpio* a*poA-Ibs* are high, which may be due to the tetraploidization of the common carp genome in evolution [22,43]. Significant growth associations were found at two SNPs in the present study (apoA-Ib.2-g.183A>T and apoA-Ib.2-g.1753C>T), and these associations were verified in two common carp populations from different river basins, suggesting the possibility of joint regulation of growth (Table 2). These two SNPs may be involved in genetic regulation of the phenotypes or in linkage disequilibrium (LD) with a nearby quantitative trait locus for growth traits [44]. Kuhnlein et al. [45] pointed out that genetic association between a polymorphism in *GH* and the egg production trait may be due to the linkage with a QTL, rather than *GH* itself. We speculated that apoA-Ib.2-g.183A>T in the intron of 5’-UTR may be involved in transcriptional regulation of *C. carpio* *apoA-Ib.2*, as suggested by Tokuhiro et al. [46]. Mutations in UTRs could affect the fate of mRNA molecules and lead to the onset of pathologies [47]. For example, Fackenthal and Olopade [48] pointed out that mutations in 5’-UTR of *BRCA1* and *BRCA2* could predict the risk of breast cancer. In this study, the locus apoA-Ib.2-g.1753C>T in exon 3 of *C. carpio* *apoA-Ib.2* is a non-synonymous mutation (from isoleucine to threonine), which may affect the structure and function of the *C. carpio* *apoA-Ib.2* [49].

## 4. Experimental Section

### 4.1. Sample Collection and Extraction

In the current study, animal procedures were based on institutional regulations and guideline for experimental animals of the Hubei Provincial Committee for animal welfare (Permit Number: 20130522-02). All efforts were made to minimize the number of animals used and the animals were treated in a humane manner.

A full-sib family of Yellow River carp was produced by artificial propagation at Henan Academy of Fishery Sciences and cultured in a muddy pond. Embryos of different stages (multi-cell stage, blastula, gastrula, optic vesicle, embryo at muscle contraction 42 h (42 h EMC), 53 h EMC, 63 h EMC, 72 h EMC, 90 h EMC) and larval carps of 6 days post-hatch (6 dph), 9 dph and 13 dph were collected. A total of fifteen progenies were sacrificed after 8 months of cultivation and divided into three groups according to their body weight (light body weight group, medium body weight group and heavy body weight group, with an average weight of 180.9, 498.3 and 835.4 g, respectively). Twelve tissues, including the brain, skin, heart, gill, spleen, eye, muscle, testis, kidney, intestine, pituitary and liver, were sampled for gene expression analysis. Individuals in the medium group were used to study tissue expression pattern of the *C.* *carpio* *apoA-Ib*, and the light and heavy groups were used for the comparative analysis of the expression levels. All embryos, larvae and tissue samples were stored in RNA safe stabilizer reagent (Omega Bio-Tek, Doraville, GA, USA) at −80 °C until RNA isolation.

Two *Cyprinus carpio* populations were generated by artificial propagation for the association analysis. One is the Yellow River carp containing 26 full-sib families, which was produced by crossing 26 males and 26 females in May 2011 at Henan Academy of Fishery Sciences. The other is the Yangtze River carp, which was generated from a mating of 5 males and 2 females in the Zhangdu Lake Fish Farm (Wuhan, China) in May 2012 and used for the verification of the growth-associated loci detected in the Yellow River carp. Two hundred individuals were randomly collected from each population, and two growth traits (BL and BW) were measured after eight months of cultivation. The formula of *K* = 100 × BW/BL^3^ was used for the calculation of Fulton’s condition factor [50].

Total RNA was extracted from embryos, larvae and tissues using the TRIZOL method (Invitrogen, Carlsbad, CA, USA). Genomic DNA was extracted from ethanol-preserved fin tissues using a standard phenol-chloroform protocol [51]. The quality of isolated RNA and DNA was evaluated by visualization on 1.5% agarose gels and by spectrophotometric analysis using a NanoDrop 2000 UV-VIS spectrophotometer (Thermo Fisher Scientific, Wilmington, DE, USA). Reverse transcription reactions were carried out to obtain total cDNA using the Reverse Transcriptase M-MLV kit (TaKaRa, Dalian, China) with oligo (dT) primer.

### 4.2. Sequence Analysis of C. carpio apoA-Ib

The amino acid sequences of ApoA-I in some vertebrates were downloaded from the National Center for Biotechnology Information (NCBI). Multiple alignments were performed with Clustal X. Twelve amino acid sequences were sectioned into different regions as described by Li et al. [1], including the 18-amino acid-long signal peptide, the 5-amino acid-long prosegment, the unrelated coding regions 1 and 2, the 33-codon block and the 11- or 22-residue repeats. The phylogenetic tree was constructed using MEGA 4.1 by applying the neighbor-joining (NJ) method [52] based on the Poisson-corrected distances with 1000 bootstraps.

### 4.3. Gene Expression Analysis by Quantitative Real-Time PCR

Due to the small difference in mRNA sequences, our pilot study showed that expression patterns of the two subtypes of *C. carpio* *apoA-Ib* were undistinguishable, and they consistently represented the overall level for *C. carpio* *apoA-Ib*. In this study, we selected one of the intron-spanning primers designed for qRT-PCR to analyze the expression level of *C. carpio* *apoA-Ib* (Table 3). The *β-actin* gene of common carp (Table 3) served as the internal control. PCR was performed in a volume of 12 μL using the StepOne^TM^ Real-Time PCR System (Applied Biosystems, Foster City, CA, USA), including 6.3 μL Power SYBR Green PCR Master Mix (Applied Biosystems), 0.25 μM of each forward and reverse primer, 1.0 μL diluted cDNA and 4.0 µL sterile distilled water. The amplification program was 95 °C for 10 min, 40 cycles of 95 °C for 15 s and 60 °C for 1 min. All samples were amplified in triplicate, and the mean values of the threshold cycle (*C*_t_) were obtained for further analysis. The relative expression levels were normalized to the quantification of *β-actin* using the 2^−ΔΔ*C*t^ method [53]. A one-way analysis of variance (ANOVA) function in the SPSS 19.0 software (SPSS Inc., Chicago, IL, USA) was applied to the data analysis, and a critical value of *p* < 0.05 was set as the criterion for statistical significance.

### 4.4. Compensatory Growth Test

Fifty fish with the same size were selected for the compensatory growth test. Before starting the test, all fish were fed three times per day for 5 days. Ten of the fish were randomly selected as the control group and fed three times per day during the entire test. The remaining forty fish were used as the experimental group to conduct the compensatory growth test. Five tissues (the pituitary, brain, liver, kidney, heart) from each fish were collected at the end of each stage: Stage 1 (starvation 0 day) with 10 fish; Stage 2 (starvation 10 days) with 10 fish; Stage 3 (starvation 20 days) with 10 fish; Stage 4 (re-feeding 10 days) with remaining 10 fish fed three times per day for 10 days after starvation 20 d. Body weight of each fish in the control group was measured at every stage, and body weight of the fish in each stage of the experiment group was measured before being slaughtered. Total RNA was extracted from all samples using the TRIZOL method (Invitrogen) and used for the qRT-PCR analysis mentioned above.

### 4.5. Identification of Polymorphisms and Association Analysis with Growth Traits

Polymorphism identification of *apoA-Ibs* was performed using 10 individuals of common carp which were collected from the Yellow River. Two pairs of PCR primers for each gene (Table 3; apoA-Ib.1-1, apoA-Ib.1-2, apoA-Ib.2-1 and apoA-Ib.2-2) were designed for SNP identification and genotyping based on the sequences of *apoA-Ib.1* and *apoA-Ib.2* of common carp (GenBank Accession Nos. KJ741859 and KJ741860). The PCR was performed in a 25-μL volume reaction on a veriti^TM^ 96-well thermal cycler (Applied Biosystems), with each reaction containing 60 ng genomic DNA, 2.5 μL 10× reaction buffer, 0.8 μL dNTP (10 mmol/L), 0.25 μM for each primer, 1.25 U Taq DNA polymerase (TaKaRa, Dalian, China) and 18 µL sterile distilled water. The amplification was realized following the thermo-profile: 5 min at 94 °C, followed by 35 cycles of 94 °C for 40 s, annealing of 60 °C for 40 s and 72 °C for 50 s and the last extension at 72 °C for 15 min. The sequences were obtained by direct sequencing of amplification products and aligned using Clustal X to identify polymorphic loci. One hundred individuals from the population of Yellow River carp were initially used for SNP genotyping. Finally, three SNPs were genotyped with the remaining 100 individuals of Yellow River carp and two of them were further genotyped in 200 individuals of Yangtze River carp, which were completed by PCR-RFLP analysis using specific restriction enzymes (Table 3).

The calculations of the genotypic and allelic frequencies, expected heterozygosity (*H*_E_), observed heterozygosity (*H*_O_) and the test for Hardy–Weinberg equilibriums (*P*_HW_) were achieved by Arlequin software Version 3.1. Association analysis between genotypes of SNPs and growth traits was performed by the general linear model (GLM) in SPSS 19.0. The model was given as follows: *Y = μ + G + e*, where *Y* is the measurement of growth traits, *μ* is the mean value of growth traits, *G* is the fixed effects of genotypes and *e* is the random residual error. The values of *p* < 0.05 and *p* < 0.01 were set as the criterion for statistically significant and extremely significant, respectively.

## 5. Conclusions

In this study, we characterized two isoforms of *apoA-Ib* in common carp, which demonstrated that ApoA-I is relatively conserved in teleost fish. Temporal and spatial expression patterns of *C. carpio* *apoA-Ib* were revealed, and marked differences in gene expression were observed between light and heavy body weight groups and before and after re-feeding in the compensatory growth test. Significant genetic associations were detected between two of the SNPs in mature mRNA of *apoA-Ib.2* and growth traits in two common carp populations. These results will help to elucidate the function and genetic effects of *apoA-Ib* on the growth traits of aquaculture fish and to apply the obtained information for gene (marker)-assisted selective breeding in common carp.

## Figures and Tables

**Table 1:** Genetic diversity based on single-nucleotide polymorphisms of a*poA-Ibs* in the test population of Yellow River carp.

Gene	Loci	Position	Genotypic Frequency		Allelic Frequency		*H_O_*	*H_E_*	*P_HW_*
*apoA-Ib.1*	g.501A>T	5′-UTR	TT	0.053	T	0.321	0.537	0.438	0.034
			AA	0.411	A	0.679			
			AT	0.537					
	g.1092A>T	Exon2	TT	0.325	T	0.575	0.525	0.492	0.648
			AA	0.163	A	0.425			
			AT	0.513					
	g.1114A>T	Exon2	TT	0.263	T	0.519	0.513	0.502	1.000
			AA	0.225	A	0.481			
			AT	0.513					
	g.1444G>A	Exon3	AA	0.673	A	0.811	0.276	0.308	0.323
			GG	0.051	G	0.189			
			AG	0.276					
	g.1500C>T	Exon3	CC	0.663	C	0.832	0.337	0.281	0.066
			CT	0.337	T	0.168			
	g.1506G>A	Exon3	AA	0.490	A	0.719	0.459	0.241	0.219
			GG	0.051	G	0.281			
			AG	0.459					
	g.1518G>A	Exon3	AA	0.010	A	0.117	0.214	0.208	1.000
			GG	0.776	G	0.883			
			AG	0.214					
	g.1569A>C	Exon3	AA	0.898	A	0.949	0.102	0.097	1.000
			AC	0.102	C	0.051			
	g.1687T>A	Exon3	TT	0.060	T	0.23	0.340	0.356	0.777
	g.1689T>C		AA	0.600	A	0.77			
	g.1693A>C		AT	0.340					
	g.1728C>T	Exon3	CC	0.663	C	0.832	0.337	0.281	0.067
			CT	0.337	T	0.168			
	g.1746C>T	Exon3	CC	0.571	C	0.658	0.173	0.452	0.000
			TT	0.255	T	0.342			
			CT	0.173					
	g.1884C>T	Exon3	CC	0.837	C	0.908	0.163	0.168	0.577
			TT	0.010	T	0.092			
			CT	0.163					
	g.1961G>C	3′-UTR	CC	0.898	C	0.949	0.102	0.097	1.000
	g.1966G>C		GC	0.102	G	0.051			
	g.1985A>G	3′-UTR	GG	0.776	G	0.888	0.224	0.200	0.602
			GA	0.224	A	0.112			
	g.1991C>T	3′-UTR	CC	0.133	C	0.352	0.439	0.459	0.667
			TT	0.429	T	0.648			
			CT	0.439					
	g.2004A>T	3′-UTR	AA	0.735	A	0.867	0.265	0.231	0.208
			AT	0.265	T	0.133			
	g.2009C>G	3'-UTR	GG	0.612	G	0.694	0.163	0.427	0.000
			CC	0.224	C	0.306			
			GC	0.163					
*apoA-Ib.2*	g.183A>T	5′-UTR	TT	0.135	T	0.422	0.573	0.490	0.140
			AA	0.292	A	0.578			
			AT	0.573					
	g.1604C>T	Exon3	CC	0.530	C	0.725	0.390	0.401	0.805
			TT	0.080	T	0.275			
			CT	0.390					
	g.1753C>T	Exon3	CC	0.380	C	0.64	0.520	0.463	0.279
			TT	0.100	T	0.36			
			CT	0.520					
	g.1767G>C	Exon3	CC	0.900	C	0.95	0.100	0.095	1.000
			CG	0.100	G	0.05			
	g.1850C>A	Exon3	CC	0.770	C	0.885	0.230	0.205	0.353
			CA	0.230	A	0.115			
	g.1862G>A	Exon3	GG	0.920	G	0.955	0.070	0.183	0.171
			GA	0.080	A	0.045			
	g.1949G>C	Exon3	GG	0.060	G	0.305	0.490	0.426	0.161
			CC	0.450	C	0.695			
			GC	0.490					
	g.2077T>G	3′-UTR	TT	0.280	T	0.565	0.570	0.494	0.155
			GG	0.150	G	0.435			
			TG	0.570					

**Table 2:** Multiple comparisons between genotypes of two SNPs and growth traits in common carp.

Loci	Populations	Genotypes	*n*	BL (cm)	BW (g)	*K*
apoA-Ib.2-g.183 A>T	Yellow River carp	AA	69	17.99 ± 0.15 ^A^	131.5 ± 2.55 ^A^	2.26 ± 0.024
		TT	25	18.59 ± 0.21 ^A,B^	141.7 ± 3.61 ^A,B^	2.20 ± 0.034
		AT	102	18.45 ± 0.14 ^B^	140.0 ± 2.36 ^B^	2.23 ± 0.022
	Yangtze River carp	AA	176	24.4 ± 2.86	378.5 ± 118.7 ^a^	2.52 ± 0.24
		AT	21	25.6 ± 2.45	440.7 ± 122.3 ^b^	2.55 ± 0.20
apoA-Ib.2-g.1753 C>T	Yellow River carp	CC	82	18.42 ± 0.14 ^a^	139.8 ± 2.44 ^A^	2.23 ± 0.023
		TT	21	18.17 ± 0.23 ^a,b^	134.6 ± 3.95 ^A,B^	2.24 ± 0.037
		CT	93	18.30 ± 0.13 ^b^	136.4 ± 2.23 ^B^	2.22 ± 0.021
	Yangtze River carp	CC	160	24.8 ± 2.68 ^A^	396.3 ± 115.7 ^A^	2.53 ± 0.24
		CT	40	23.4 ± 3.14 ^B^	341.3 ± 127.3 ^B^	2.56 ± 0.23

^a,b^ The different superscript lowercase letters within a column mean significant difference, *p* < 0.05; ^A,B^ the different superscript uppercase letters within a column mean extremely significant difference, *p* < 0.01; BL, body length; BW, body weight; *K*, Fulton’s condition factor.

**Table 3:** Primers used in this study.

Primer Name	Primer Sequence (Forward)	Primer Sequence (Reverse)	Usage
apoA-Ib-qPCR-1	GAGCCGCCGTCGCAGGTGG	GAAGCCGTTCTGAAAGTAGCC	qRT-PCR
β-actin-qPCR	TATCCTATTGAGCACGGTATTG	CCTGTTGGCTTTGGGATTC	qRT-PCR
apoA-Ib.1-1	TCTCTTCCCAGACCAGCTACAGTAAC	ATCTAAACATCCCTTTGACC	SNP identification and genotyping
apoA-Ib.1-2	GCTCTTGCTTGTTTATGCC	TTGCGAGGATGTGTTAGTGT	SNP identification and genotyping
apoA-Ib.2-1	TCTCTTCCCAGACCAGCTACAGTAAC	GCTGATGTTTTGACAGTGTTGAGAG	SNP identification and genotyping
apoA-Ib.2-2	ACATCAGCATTGTTGTCTTC	GAGTGTATTGTGAGCAGGTGTG	SNP identification and genotyping
apoA-Ib.1-RFLP-1	TCTCTTCCCAGACCAGCTACAGTAAC	TTTTTTTTGACGGTGTGGAGAGGATT	genotyping locus apoA-Ib.1-g.501 A>T
apoA-Ib.1-RFLP-2	GCAGTTTTAGTTCTTTTATTGG	TTGCGAGGATGTGTTAGTGT	genotyping locus apoA-Ib.1-g.1693 A>C
apoA-Ib.2-RFLP-1	ACATCAGCATTGTTGTCTTC	GAGCCTTGGTTCTTACTTG	genotyping locus apoA-Ib.2-g.183 A>T
apoA-Ib.2-RFLP-2	ATGGCTACTTTCAGAACGGC	GAGTGTATTGTGAGCAGGTGTG	genotyping locus apoA-Ib.2-g.1753 C>T

## Supplementary Materials

Supplementary materials can be found at www.mdpi.com/1422-0067/17/9/1569/s1.

## References

[1] Li W.H., Tanimura M., Luo C.C., Datta S., Chan L. (1988). The apolipoprotein multigene family—Biosynthesis, structure, structure-function relationships, and evolution. J. Lipid Res..

[2] De Smet H., Blust R., Moens L. (1998). Absence of albumin in the plasma of the common carp *Cyprinus carpio*: Binding of fatty acids to high density lipoprotein. Fish Physiol. Biochem..

[3] Bolanos-Garcia V.M., Miguel R.N. (2003). On the structure and function of apolipoproteins: More than a family of lipid-binding proteins. Prog. Biophys. Mol. Biol..

[4] Srinivas R.V., Venkatachalapathi Y.V., Rui Z., Owens R.J., Gupta K.B., Srinivas S.K., Anantharamaiah G.M., Segrest J.P., Compans R.W. (1991). Inhibition of virus-induced cell-fusion by apolipoprotein A-I and its amphipathic peptide analogs. J. Cell. Biochem..

[5] Tada N., Sakamoto T., Kagami A., Mochizuki K., Kurosaka K. (1993). Antimicrobial activity of lipoprotein particles containing apolipoprotein-A-l. Mol. Cell. Biochem..

[6] Burger D., Dayer J. (2002). High-density lipoprotein-associated apolipoprotein AI: The missing link between infection and chronic inflammation?. Autoimmun. Rev..

[7] Ma J., Liao X.-L., Lou B., Wu M.-P. (2004). Role of apolipoprotein AI in protecting against endotoxin toxicity. Acta Biochim. Biophys. Sin..

[8] Brewer H., Fairwell T., LaRue A., Ronan R., Houser A., Bronzert T. (1978). The amino acid sequence of human ApoA-I, an apolipoprotein isolated from high density lipoproteins. Biochem. Biophys. Res. Commun..

[9] Ferrari S., Drusiani E., Calandra S., Tarugi P. (1986). Isolation of a cDNA clone for chicken intestinal apolipoprotein AI and its use for detecting apoAI mRNA expression in several chicken tissues. Gene.

[10] Chan L. (1989). The apolipoprotein multigene family—Structure, expression, evolution, and molecular-genetics. Klin. Wochenschr..

[11] Watanabe T. (1982). Lipid nutrition in fish. Comp. Biochem. Physiol. B Comp. Biochem..

[12] Kondo H., Morinaga K., Misaki R., Nakaya M., Watabe S. (2005). Characterization of the pufferfish *Takifugu rubripes* apolipoprotein multigene family. Gene.

[13] Powell R., Higgins D.G., Wolff J., Byrnes L., Stack M., Sharp P.M., Gannon F. (1991). The salmon gene encoding apolipoprotein-A-I—cDNA sequence, sissue sxpression and svolution. Gene.

[14] Llewellyn L., Ramsurn V.P., Wigham T., Sweeney G.E., Power D.M. (1998). Cloning, characterisation and expression of the apolipoprotein A-I gene in the sea bream (*Sparus aurata*). Biochim. Biophys. Acta (BBA)-Gene Struct. Exp..

[15] Kondo H., Kawazoe I., Nakaya M., Kikuchi K., Aida K., Watabe S. (2001). The novel sequences of major plasma apolipoproteins in the eel *Anguilla japonica*. Biochim. Biophys. Acta (BBA)-Mol. Cell Biol. Lipids.

[16] Concha M.I., Lopez R., Villanueva J., Baez N., Amthauer R. (2005). Undetectable apolipoprotein A-I gene expression suggests an unusual mechanism of dietary lipid mobilisation in the intestine of *Cyprinus carpio*. J. Exp. Biol..

[17] Villarroel F., Bastias A., Casado A., Amthauer R., Concha M.I. (2007). Apolipoprotein A-I, an antimicrobial protein in *Oncorhynchus mykiss*: Evaluation of its expression in primary defence barriers and plasma levels in sick and healthy fish. Fish Shellfish Immunol..

[18] Dietrich M.A., Adamek M., Bilinska B., Hejmej A., Steinhagen D., Ciereszko A. (2014). Characterization, expression and antibacterial properties of apolipoproteins A from carp (*Cyprinus carpio* L.) seminal plasma. Fish Shellfish Immunol..

[19] Concha M.I., Molina S.A., Oyarzún C., Villanueva J., Amthauer R. (2003). Local expression of apolipoprotein AI gene and a possible role for HDL in primary defence in the carp skin. Fish Shellfish Immunol..

[20] Liu X., Bai J., Yu L., Li S. (2012). SNPs screening of 3′UTR in *apoprotein A1-1* gene and its association with growth traits in grass carp. J. Dalian Ocean Univ..

[21] Bostock J., McAndrew B., Richards R., Jauncey K., Telfer T., Lorenzen K., Little D., Ross L., Handisyde N., Gatward I. (2010). Aquaculture: Global status and trends. Philos. Trans. R. Soc. B.

[22] Wang J.T., Li J.T., Zhang X.F., Sun X.W. (2012). Transcriptome analysis reveals the time of the fourth round of genome duplication in common carp (*Cyprinus carpio*). BMC Genom..

[23] Xu P., Zhang X.F., Wang X.M., Li J.T., Liu G.M., Kuang Y.Y., Xu J., Zheng X.H., Ren L.F., Wang G.L. (2014). Genome sequence and genetic diversity of the common carp, *Cyprinus carpio*. Nat. Genet..

[24] Otis J.P., Zeituni E.M., Thierer J.H., Anderson J.L., Brown A.C., Boehm E.D., Cerchione D.M., Ceasrine A.M., Avraham-Davidi I., Tempelhof H. (2015). Zebrafish as a model for apolipoprotein biology: Comprehensive expression analysis and a role for ApoA-IV in regulating food intake. Dis. Models Mech..

[25] Choudhury M., Oku T., Yamada S., Komatsu M., Kudoh K., Itakura T., Ando S. (2011). Isolation and characterization of some novel genes of the apolipoprotein A-I family in Japanese eel, *Anguilla japonica*. Cent. Eur. J. Biol..

[26] Delcuve G.P., Sun J.M., Davie J.R. (1992). Expression of rainbow-trout apolipoprotein-A-I genes in liver and hepatocellular-carcinoma. J. Lipid Res..

[27] Kim K.Y., Cho Y.S., Bang I.C., Nam Y.K. (2009). Isolation and characterization of the apolipoprotein multigene family in *Hemibarbus mylodon* (Teleostei: Cypriniformes). Comp. Biochem. Phys. B.

[28] Furlaneto C.J., Ribeiro F.P., Hatanaka E., Souza G.M., Cassatella M.A., Campa A. (2002). Apolipoproteins A-I and A-II downregulate neutrophil functions. Lipids.

[29] Lewis G.F., Rader D.J. (2005). New insights into the regulation of HDL metabolism and reverse cholesterol transport. Circ. Res..

[30] Magnadóttir B. (2006). Innate immunity of fish (overview). Fish Shellfish Immunol..

[31] Fang L.H., Choi S.H., Baek J.S., Liu C., Almazan F., Ulrich F., Wiesner P., Taleb A., Deer E., Pattison J. (2013). Control of angiogenesis by AIBP-mediated cholesterol efflux. Nature.

[32] Seo J., Yun C.O., Kwon O.J., Choi E.J., Song J.Y., Choi I., Cho K.H. (2012). A proteoliposome containing apolipoprotein A-I mutant (V156K) enhances rapid tumor regression activity of human origin oncolytic adenovirus in tumor-bearing zebrafish and mice. Mol. Cells.

[33] Babin P.J., Thisse C., Durliat M., Andre M., Akimenko M.A., Thisse B. (1997). Both apolipoprotein E and AI genes are present in a nonmammalian vertebrate and are highly expressed during embryonic development. Proc. Natl. Acad. Sci. USA.

[34] Cunha I., Galante-Oliveira S., Rocha E., Urbatzka R., Castro L.F.C. (2015). Expression of intercellular lipid transport and cholesterol metabolism genes in eggs and early larvae stages of turbot, *Scophthalmus maximus*, a marine aquaculture species. Mar. Biol..

[35] Adlouni A., Ghalim N., Saile R., Hda N., Parra H.J., Benslimane A. (1998). Beneficial effect on serum apo AI, apo B and Lp AI levels of Ramadan fasting. Clin. Chim. Acta.

[36] Akanji A.O., Mojiminiyi O.A., Abdella N. (2000). Beneficial changes in serum apo A-1 and its ratio to apo B and HDL in stable hyperlipidaemic subjects after Ramadan fasting in Kuwait. Eur. J. Clin. Nutr..

[37] Afolabi P.R., Jahoor F., Jackson A.A., Stubbs J., Johnstone A.M., Faber P., Lobley G., Gibney E., Elia M. (2007). The effect of total starvation and very low energy diet in lean men on kinetics of whole body protein and five hepatic secretory proteins. Am. J. Physiol.-Endocrinol. Metab..

[38] De-Santis C., Jerry D.R. (2007). Candidate growth genes in finfish—Where should we be looking?. Aquaculture.

[39] Tong J.G., Sun X.W. (2015). Genetic and genomic analyses for economically important traits and their applications in molecular breeding of cultured fish. Sci. China Life Sci..

[40] Sun Y.H., Yu X.M., Tong J.G. (2012). Polymorphisms in myostatin gene and associations with growth traits in the common carp (*Cyprinus carpio* L.). Int. J. Mol. Sci..

[41] Feng X., Yu X.M., Tong J.G. (2014). Novel single nucleotide polymorphisms of the insulin-like growth factor-I gene and their associations with growth traits in common carp (*Cyprinus carpio* L.). Int. J. Mol. Sci..

[42] Feng X., Yu X.M., Pang M.X., Liu H.Y., Tong J.G. (2015). Molecular characterization and expression of three preprosomatostatin genes and their association with growth in common carp (*Cyprinus carpio*). Comp. Biochem. Phys. B.

[43] David L., Blum S., Feldman M.W., Lavi U., Hillel J. (2003). Recent duplication of the, common carp (*Cyprinus carpio* L.) genome as revealed by analyses of microsatellite loci. Mol. Biol. Evol..

[44] Lynch M., Walsh B. (1998). Genetics and Analysis of Quantitative Traits.

[45] Kuhnlein U., Ni L., Weigend S., Gavora J.S., Fairfull W., Zadworny D. (1997). DNA polymorphisms in the chicken growth hormone gene: Response to selection for disease resistance and association with egg production. Anim. Genet..

[46] Tokuhiro S., Yamada R., Chang X.T., Suzuki A., Kochi Y., Sawada T., Suzuki M., Nagasaki M., Ohtsuki M., Ono M. (2003). An intronic SNP in a *RUNX1* binding site of *SLC22A4*, encoding an organic cation transporter, is associated with rheumatoid arthritis. Nat. Genet..

[47] Pesole G., Mignone F., Gissi C., Grillo G., Licciulli F., Liuni S. (2001). Structural and functional features of eukaryotic mRNA untranslated regions. Gene.

[48] Fackenthal J.D., Olopade O.I. (2007). Breast cancer risk associated with *BRCA1* and *BRCA2* in diverse populations. Nat. Rev. Cancer.

[49] Ramensky V., Bork P., Sunyaev S. (2002). Human non-synonymous SNPs: Server and survey. Nucleic Acids Res..

[50] Nash R.D.M., Valencia A.H., Geffen A.J. (2006). The origin of Fulton’s condition factor—Setting the record straight. Fisheries.

[51] Sambrook J., Russell D. (2001). Molecular Cloning: A Laboratory Manual.

[52] Saitou N., Nei M. (1987). The neighbor-joining method—A new method for reconstructing phylogenetic trees. Mol. Biol. Evol..

[53] Livak K.J., Schmittgen T.D. (2001). Analysis of relative gene expression data using real-time quantitative PCR and the 2^−ΔΔ*C*t^ method. Methods.

