# Supplementary material for: Molecular Characterization and Growth Association of Two Apolipoprotein A-Ib Genes in Common Carp (Cyprinus carpio)
Source: Int J Mol Sci. 2016 Sep 16;17(9):1569. doi: 10.3390/ijms17091569 (PMC5037836; doi:10.3390/ijms17091569)
Supplement: Supplementary file 1 [file ijms-17-01569-s001.pdf]

Supplementary Materials: Molecular Characterization and Growth Association of Two Apolipoprotein A-Ib Genes in Common Carp (*Cyprinus carpio*)

Xinhua Wang, Xiaomu Yu and Jingou Tong

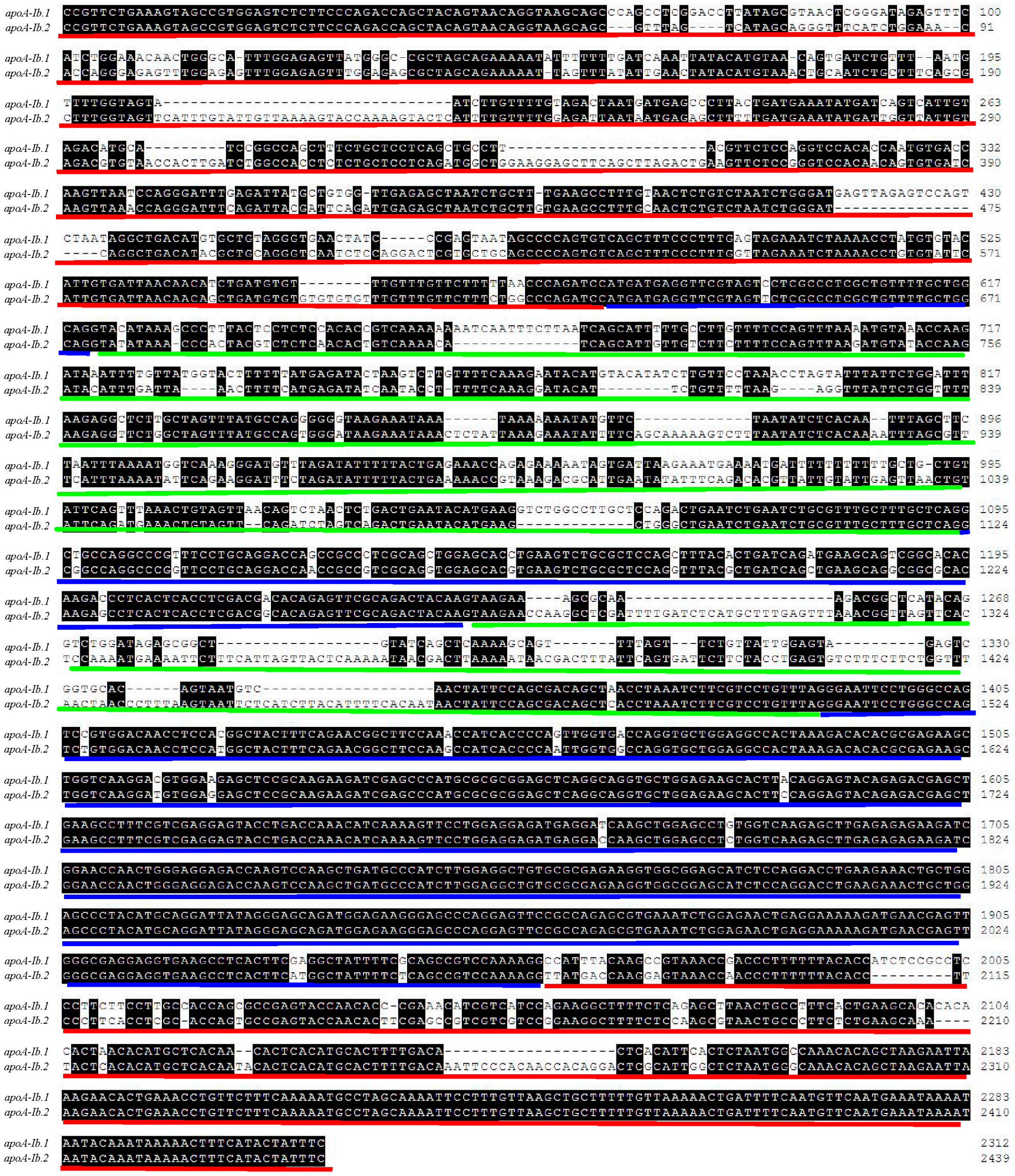

Figure S1. Comparison of the nucleotide sequences of two *apoA-Ibs* in common carp. Identity is covered with black color. The UTRs, exons and introns were underlined with red color, blue color and green color respectively.

Table S1. Information of 63 amino acids sequences in phylogenetic analysis.

Species	Protein	GenBank Accession No.	The Number of Amino Acids
Ailuropoda melanoleuca	ApoA-I	XP_002919539	266
Alligator sinensis	ApoA-I	XP_006030393	259
Anguilla japonica	ApoA-I	AB219180	263
Aristicluthys nobilia	ApoA-I	unpublished result	257
Astyanax mexicanus	ApoA-I	XM_007239417	260
Bos taurus	ApoA-I	NP_776667	265
Callithrix jacchus	ApoA-I	XP_009005100	263
Callorhinchus milii	ApoA-I	XP_007905508	261
Canis lupus familiaris	ApoA-I	XP_013968873	266
Channa striata	ApoA-I	AIL82447	273
Cirrhinus molitorella	ApoA-I	GU139545	257
Clupea harengus	ApoA-I	XM_012828452	262
Columba livia	ApoA-I	XP_005500766	264
Coturnix japonica	ApoA-I	XP_015739386	264
Cynoglossus semilaevis	ApoA-I	XP_008307644	268
Cyprinus carpio	ApoA-I	KF268349	257
Cyprinus carpio	ApoA-Ib.1	KJ741859	257
Cyprinus carpio	ApoA-Ib.2	KJ741860	257
Danio rerio	ApoA-Ia	NP_571203	262
Danio rerio	ApoA-Ib	NP_001093614	257
Equus asinus	ApoA-I	XP_014711022	266
Erinaceus europaeus	ApoA-I	XP_007523042	265
Esox lucius	ApoA-I	XP_010895444	260
Felis catus	ApoA-I	XP_011284668	266
Gallus gallus	ApoA-I	NP_990856	264
Gekko japonicus	ApoA-I	XP_015283537	272
Gobiocypris rarus	ApoA-I	ABY47600	256
Hemibarbus mylodon	ApoA-I.1	FJ170104	257
Hemibarbus mylodon	ApoA-I.2	FJ170105	262
Homo sapiens	ApoA-I	NP_000030	267
Hypophthalmichthys molitrix	ApoA-I	ADF97611	257
Ictalurus furcatus	ApoA-I	NP_001187398	258
Ictalurus punctatus	ApoA-I	NP_001187398	258
Labeo rohita	ApoA-I	KC934748	256
Latimeria chalumnae	ApoA-I	XP_005987291	237
Leptonychotes weddellii	ApoA-I	XP_006743516	266
Lipotes vexillifer	ApoA-I	XP_007461913	265
Macaca mulatta	ApoA-I	XP_001090774	267
Morone saxatilis	ApoA-I	ACH90228	190
Mus musculus	ApoA-I	NP_033822	264
Notothenia coriiceps	ApoA-I	XP_010792180	263
Oncorhynchus mykiss	ApoA-I.1	NP_001117719	262
Oncorhynchus mykiss	ApoA-I.2	NP_001117720	262
Orcinus orca	ApoA-I	XP_004273413	265
Oreochromis niloticus	ApoA-I	CCF55060	263
Oryctolagus cuniculus	ApoA-I	NP_001095157	265
Pan paniscus	ApoA-I	XP_003820129	267
Pelodiscus sinensis	ApoA-I	XP_006121737	262

Table S1. *Cont.*

Species	Protein	GenBank Accession No.	The Number of Amino Acids
Physeter catodon	ApoA-I	XP_007117218	265
Platichthys flesus	ApoA-I	CAH59609	263
Poecilia formosa	ApoA-I	XP_007576027	263
Poecilia reticulata	ApoA-I	XP_008424299	263
Python bivittatus	ApoA-I	XP_015747026	261
Rattus norvegicus	ApoA-I	NP_036870.1	259
Rhinopithecus roxellana	ApoA-I	XP_010369999	267
Salmo salar	ApoA-I	NP_001134612	262
Salmo trutta	ApoA-I	Q91488	262
Serinus canaria	ApoA-I	XP_009096256	264
Sinocyclocheilus anshuiensis	ApoA-I.1	XM_016454056	257
Sinocyclocheilus anshuiensis	ApoA-I.2	XM_016531543	257
Sparus aurata	ApoA-I	O42175	260
Sus scrofa	ApoA-I	NP_999563	264
Xenopus laevis	ApoA-I	NP_001079732	260
